# Severe A(H1N1)pdm09 influenza acute encephalopathy outbreak in children in Tuscany, Italy, December 2023 to January 2024

**DOI:** 10.2807/1560-7917.ES.2024.29.17.2400199

**Published:** 2024-04-25

**Authors:** Luca Bartolini, Silvia Ricci, Chiara Azzari, Maria Moriondo, Francesco Nieddu, Manuela L’Erario, Zaccaria Ricci, Gabriele Simonini, Marzia Mortilla, Giuseppe Indolfi, Carlotta Montagnani, Elena Chiappini, Luisa Galli, Renzo Guerrini

**Affiliations:** 1 Neuroscience Department, Meyer Children’s Hospital IRCCS, Florence, Italy; 2 Department of Neuroscience, Psychology, Pharmacology and Child Health (NEUROFARBA), University of Florence, Florence, Italy; 3 Pediatric Immunology Unit, Meyer Children’s Hospital IRCCS, Florence, Italy; 4 Department of Health Sciences, University of Florence, Florence, Italy; 5 Pediatric Intensive Care Unit, Meyer Children’s Hospital IRCCS, Florence, Italy; 6 Rheumatology UNIT, ERN ReCONNET Center, Meyer Children’s Hospital IRCCS, Florence, Italy; 7 Emergency Radiology Unit, Meyer Children’s Hospital IRCCS, Florence, Italy; 8 Pediatrics and Liver Unit, Meyer Children’s Hospital IRCCS, Florence, Italy; 9 Pediatric Infectious Diseases Unit, Meyer Children’s Hospital IRCCS, Florence, Italy

**Keywords:** H1N1, Encephalopathy, Italy, Neurological, Children, Italy, air-borne infections, viral infections, influenza, influenza virus, vaccines and immunisation, clinic

## Abstract

A severe outbreak of influenza A(H1N1pdm09) infection in seven children (median age: 52 months) occurred between December 2023 and January 2024 in Tuscany, Italy. Clinical presentation ranged from milder encephalopathy to acute necrotizing encephalopathy (ANE) with coma and multiorgan failure; one child died. This report raises awareness for clinicians to identify and treat early acute encephalopathy caused by H1N1 influenza and serves as a reminder of severe presentations of influenza in young children and the importance of vaccination.

Influenza infection in young children can present with severe complications, especially during the peak season [1]. The risk of severe outcome can be exacerbated by low rates of influenza vaccination in children [2]. Here, we describe an outbreak of influenza A(H1N1)pdm09 with severe neurological involvement in seven children admitted between December 2023 and January 2024 to Meyer Children’s Hospital (Florence, Italy), a tertiary care referral centre in Tuscany with a catchment area of ca 600,000 children.

## Clinical characteristics

**Figure 1:**
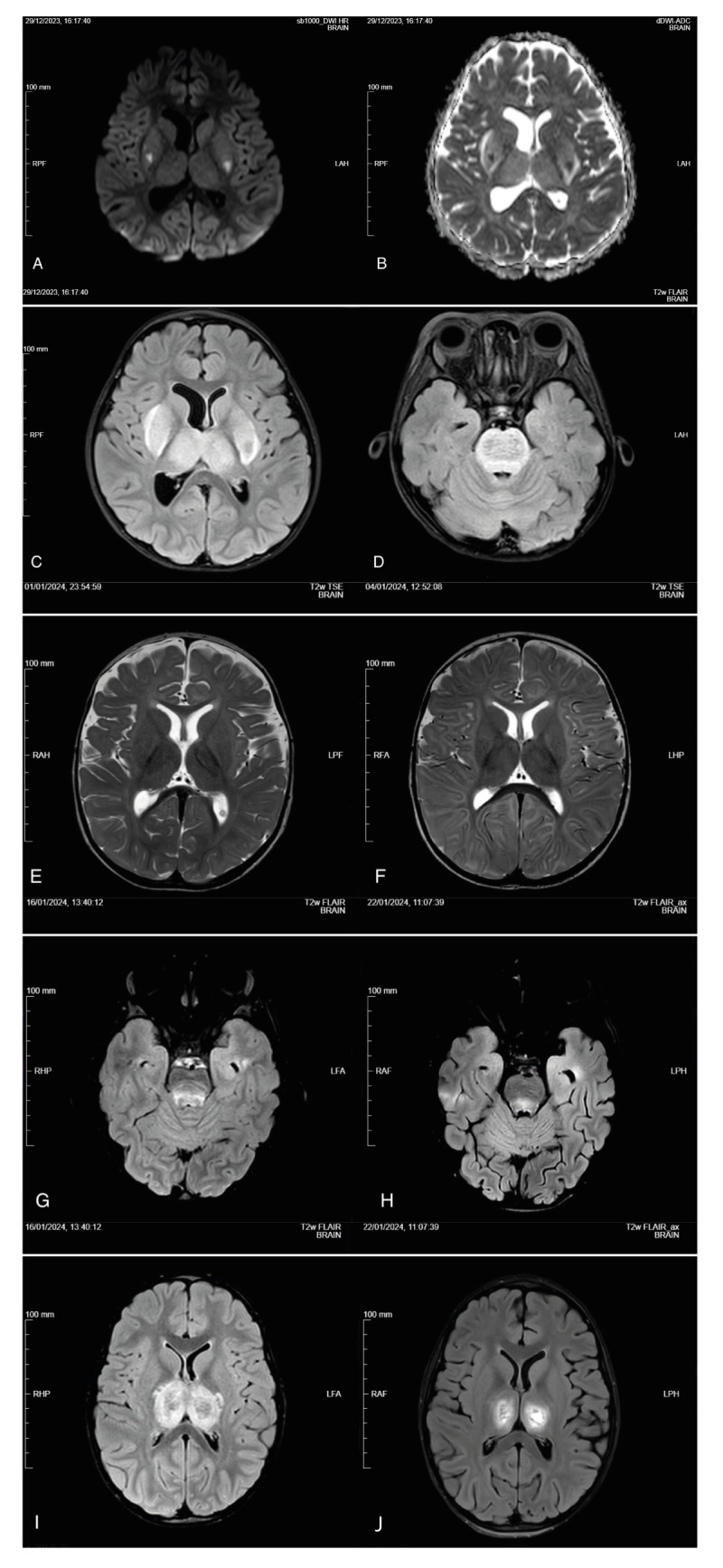
Brain magnetic resonance imaging in children with influenza A(H1N1)pdm09 acute necrotizing encephalopathy admitted to Meyer Children’s Hospital, Florence, Italy, December 2023–January 2024 (n = 3)

ADC: apparent diffusion coefficient; FLAIR: fluid attenuated inversion recovery; MRI: magnetic resonance imaging; PICU: paediatric intensive care unit.

All seven patients (median age: 52 months (interquartile range (IQR): 25–70), n = 5 males) presented with febrile illness and encephalopathy. Five children were previously healthy, while two had experienced seizures in the past. Four patients were directly admitted to the paediatric intensive care unit (PICU), and three were admitted to the hospital wards. Three PICU patients were diagnosed with acute necrotizing encephalopathy (ANE) [3]. Clinical characteristics are summarised in Table 1 and brain magnetic resonance imaging (MRI) findings are shown in Figure 1.

**Table 1:** Demographic and clinical characteristics of children admitted to Meyer Children’s Hospital for influenza A(H1N1)pdm09 infection with neurological involvement, Florence, Italy, December 2023–January 2024 (n = 7)

Patient	Age (months)	Presentation (days after fever onset)	Video EEG (days after fever onset)	Brain MRI (days after fever onset)	Antiseizure medications	Immunomodulator (days of treatment)	Oseltamivir (days of fever when started)	Relevant laboratory results	Outcome (days after fever onset)
PICU 1	52	Coagulopathy (2); multiorgan failure (3); encephalopathy (4)	Moderate slow (4); suppression (5)	Diffuse basal ganglia, thalami, brainstem injury (4)	None	MTP (2)	Yes (4)	PLT: 23x10^9^/L, AST: 11,291 IU/L, ALT: 4,398 IU/L, Cr: 3.49 mg/dL, INR: 2.89, aPTT: 106 s, CRP: 3.74 mg/dL, PCT: 290.0 ng/mL, Fib: 128 mg/dL	Death (5)
PICU 2	11	Febrile status epilepticus (1)	Ictal-interictal continuum (1–3); burst suppression (4–7)	Negative (2); diffuse white matter, thalami injury (4)	DZP, MDZ, LCM, PHT	DEX (1), MTP (5), IVIg (3), ANK (7), TOC (1)	Yes (1)	AST: 2,968 IU/L, ALT: 2,212 IU/L, Fib: 166 mg/dL, DD: 24,382 ug/L, INR: 2.05, aPTT: 53 s, CRP: 1.81 mg/dL, PCT: 42.6 ng/mL, CSF WBC: 4x1/uL Glu: 105 mg/dL, TP: 35 mg/dL	Gastrostomy tube (no tracheostomy), minimally conscious state, autonomic storms, spastic quadriplegia (63)
PICU 3	77	Encephalopathy (1)	Mild slow (1–2); frontal discharges (6)	Bilateral temporal, thalami, pons injury (1); evolution of injury (7)	CBZ	MTP (5), IVIg (3), TOC (1), PLEX (3)	Yes (1)	AST: 64 IU/L, ALT: 43 IU/L, CRP: 6.06 mg/dL, PCT: 51.7 ng/mL, CSF WBC^a^: 35x1/uL, Glu: 58 mg/dL, TP: 79 mg/dL	Mild dyspraxia (48)
PICU 4	86	Complex febrile seizures (3)	Moderate slow (3–4)	N/A	MDZ, PHT, CLB	None	Yes (3)	N/A	Full recovery (8)
Wards 1	63	Complex febrile seizures (2)	Mild slow (1)	Mild bilateral post white matter hyper (5)	CLO	None	Yes (2)	N/A	Full recovery (6)
Wards 2	16	Cough (1); febrile status epilepticus (2)	Focal bilateral occipital slow (1); normal (8)	Negative (4)	DZP, MDZ, PHT	DEX (3)	Yes (2)	CSF WBC: 1x1/uL, Glu: 73 mg/dL, TP: 11 mg/dL	Full recovery (6)
Wards 3	34	Cough (1); generalised weakness, refusal to walk, encephalopathy (4)	Mild encephalopathy (4); normal (9)	Negative (11)	None	None	No	CSF WBC: 1x1/uL, Glu: 50 mg/dL, TP: 14 mg/dL	Full recovery (7)

aPTT: activated partial thromboplastin time; ALT: alanine aminotransferase; ANK: anakinra; AST: aspartate aminotransferase; CBZ: carbamazepine; CLB: clobazam; CLO: clonazepam; Cr: creatinine; CRP: C-reactive protein; CSF: cerebrospinal fluid; DD: d-dimer; DEX: dexamethasone; DZP: diazepam; EEG: electroencephalogram; Fib: fibrinogen; Glu: glucose; INR: international normalised ratio; IVIg: intravenous immunoglobulin; LCM: lacosamide; MDZ: midazolam; MRI: magnetic resonance imaging; MTP: methylprednisolone; N/A: not applicable; PCT: procalcitonin; PHT: phenytoin; PLEX: plasma exchange; PLT: platelets; s: seconds; TOC: tocilizumab; TP: total protein; WBC: white blood cell.

Normal ranges for the laboratory results are as follows: ALT (5–19 IU/L), aPTT (29–38 s), AST (5–41 IU/L), Cr (0.30–0.50 mg/dL), CRP (0–0.50 mg/dL), CSF Glu (50–80 mg/dL), CSF TP (20–50 mg/dL), CSF WBC (< 10x1/uL), DD (< 500 ug/L), Fib (200–400 mg/dL), INR (0.92–1.14), PCT (< 0.5 ng/mL), PLT (210–590x10^9^/L),

^a^ Traumatic tap from needle insertion, with bleeding into the subarachnoid space, artificially increasing the white blood cell count.

A–D: MRI brain images from patient PICU 1. Diffusion weighted imaging (DWI) sequence (A) and ADC map (B) highlighting restricted diffusion in the putamen bilaterally. T2-FLAIR image showing oedema and hyperintense signal involving the thalami, putamen and caudate nuclei (C) and brainstem (D).

E–F: MRI brain images from patient PICU 2: T2-weighted images on the initial MRI (E) and 60 h later (F) showing progression from normal myelination pattern to diffuse white matter injury.

G–J: MRI brain images from patient PICU 3: T2-FLAIR images showing diffusely hyperintense signal in the midbrain and bilateral temporal lobes on the initial MRI (G) and 6 days later (H), and in the thalami on the initial MRI (I) and 6 days later (J).

None of the children had been vaccinated for influenza. Six of seven patients received oral oseltamivir [4] for 5–12 days upon admission. 

## Immunomodulatory treatment and outcome in severe cases

A previously healthy child (PICU 1) was transferred to our PICU on the fourth day of fever because of liver failure and severe coagulopathy with spontaneous gastrointestinal bleeding and multiorgan failure with rapid neurological deterioration. The patient was given high dose methylprednisolone (30 mg/kg once daily) and received only two doses before the child died on the fifth day of illness.

A previously healthy child (PICU 2) was transferred to our PICU because of convulsive febrile status epilepticus. The child was intubated and put on a midazolam drip to treat ictal-interictal continuum/non-convulsive status epilepticus. Immunomodulation started at 48 h of illness and consisted of dexamethasone 1 mg/kg/day for 2 days, high dose methylprednisolone (30 mg/kg once daily) for 5 days, intravenous immunoglobulin (IVIg) 1 g/kg/day for 3 days, anakinra 10 mg/kg/day for 7 days, and one dose of tocilizumab 9 mg/kg. The child was extubated on day 7 of mechanical ventilation and required gastrostomy tube placement, but no tracheostomy was necessary. Neurological examination on the 63^rd^ day of illness showed a severe outcome with minimally conscious state, autonomic storms, and severe dystonic spastic quadriplegia.

A previously healthy child (PICU 3) was admitted to our PICU because of encephalopathy after 2 days of fever. All immunomodulatory drugs were started within 24 h of illness and consisted of high dose methylprednisolone (30 mg/kg once daily) for 5 days, followed by an oral taper over 1 month, IVIg 1 g/kg/day for 3 days, plasma exchange every other day for 3 sessions, and tocilizumab (9 mg/kg/dose) once weekly for 1 month. The child was extubated after 6 days of mechanical ventilation. Neurological examination on the 48^th^ day of illness showed mild dyspraxia as the only manifestation, highlighting good clinical outcome.

The fourth child (PICU 4) admitted to the PICU was extubated and transferred out of the PICU in less than 48 h. No immunomodulation was pursued. The child made a full recovery.

Two patients admitted to the wards did not receive immunomodulatory treatment; the third patient was given dexamethasone 1 mg/kg/day for 3 days. All three children experienced full recovery before discharge.

## Genotyping and epidemiological data

According to our institutional surveillance protocol, all children admitted with fever and respiratory or neurological symptoms are tested for viral infection. A nasopharyngeal swab taken from all patients tested positive for influenza A(H1N1)pdm09. Viral PCR was negative in the cerebrospinal fluid (CSF) of the four patients who underwent a lumbar puncture. Patients were tested for other respiratory viruses: influenza A(H1N1), influenza A(H3N2), influenza B, respiratory syncytial virus (RSV), rhinovirus, adenovirus, parainfluenza virus types 1–3, metapneumovirus, SARS-CoV-2 and bocavirus; no co-infections were identified. 

Sequencing of the influenza A(H1N1)pdm09 strains from swab samples collected from the three most severe PICU patients showed that all strains belonged to clade 6B.1A.5a.2a. We did not identify any amino acid substitutions of particular interest in patients with ANE (Table 2).

**Table 2:** Genetic characterisation of the influenza A(H1N1)pdm09 strains from the most severely affected children admitted to Meyer Children’s Hospital, Florence, Italy, December 2023–January 2024 (n = 3)

Patient	Virus lineage	Clade	Identified amino acid substitutions on haemagglutinin (HA) gene from PB2A/Victoria/4897/2022(H1N1)
PICU 1	FluA(H1N1)pdm09	6B.1A.5a.2a	N55D, T137A, S154P, R159K, K186Q, A233T, R240Q, E277D, A294T, D373E, H468N, K497R
PICU 2	FluA(H1N1)pdm09	6B.1A.5a.2a	R159K, A233T, R240Q, E277D, A294T, D373E, H416R, H468N, I550L
PICU 3	FluA(H1N1)pdm09	6B.1A.5a.2a	T137A, S154P, R159K, K186Q, A233T, R240Q, E277D, A294T, D373E, H468N

All mutations are not known to alter the virulence of the virus, cause strong drug resistance or reverse the effects of the premature STOP codon in the PB1-F2 gene of pandemic H1N1 (https://flusurver.bii.a-star.edu.sg).

**Figure 2:**
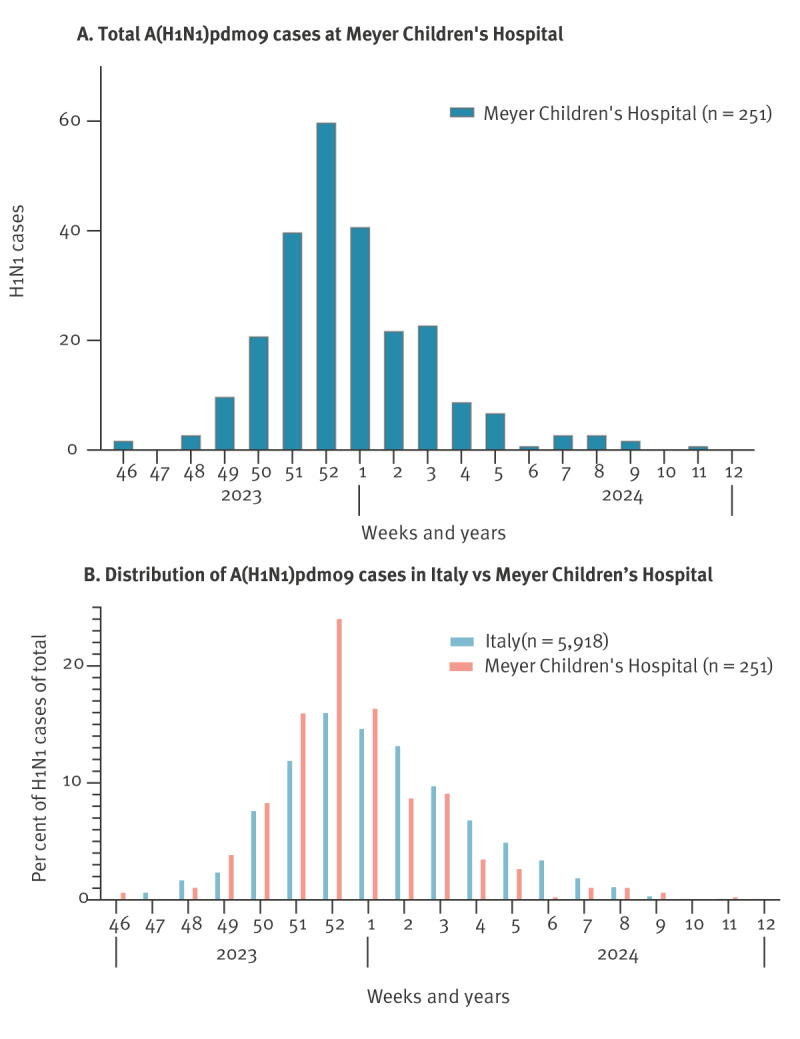
Influenza A(H1N1)pdm09 institutional surveillance data, Meyer Children’s Hospital, Florence, Italy, 2023/2024 season

Epidemiological data recorded at our institution from 1 November 2023 to 20 March 2024 for children admitted with fever and respiratory or neurological symptoms revealed 251 A(H1N1)pdm09-positive nasopharyngeal swabs (Figure 2), of which 226 were admitted during the study period between week 49 2023 and week 4 2024.

B. The per cent was calculated as weekly cases/total cases using data from Italy and from the hospital. The source for Italy data: https://respivirnet.iss.it/pagine/rapportoInflunet.aspx

## Discussion

In the post-COVID-19 pandemic seasons, influenza-like illness (ILI) incidence has increased in the European Union/European Economic Area (EU/EEA) [5]. In Italy, during the 2022/23 season, ILI peaked early, during the week ending with 27 November 2022, driven predominantly by influenza A (96.3% H3N2) and RSV [6]. The 2023/24 season has been characterised by high intensity activity, a later ILI peak between week 49 2023 and weeks 2–3 2024 and a dominance of A(H1N1)pdm09 viruses. These observations reflect the ILI peak identified through molecular surveillance in our institution, with A(H1N1)pdm09 reaching 92.4% of circulating viruses during week 48 [7]. The 6B.1A.5a.2a clade starting from week 40 of 2023 was the most prevalent type A virus [5]. At the beginning of January 2024, the United States (US) Centers for Disease Control and Prevention (CDC) raised an alert on severe influenza illness including deaths among children in the US, mainly by influenza A viruses. However, no subtyping was performed, and no description of symptoms or neurological involvement is available [8]. 

Paediatric mortality data in Italy for the 2023/24 season are not yet available. Our institutional data show that influenza cases with severe neurological involvement comprise 3% (7/226) of all children admitted during the study period for fever associated with respiratory or neurological symptoms. Because our institution used a different surveillance protocol after the COVID-19 pandemic, we are not able to compare the present data with pre-pandemic data concerning neurological involvement in children admitted for ILI. However, no case of ANE was recorded at least since 2017.

Based on a comparison between the genotype of identified virus and PB2A/Victoria/4897/2022(H1N1) available on the NEXTCLADE dataset (https://clades.nextstrain.org), we did not identify any amino acid substitutions of particular interest (in PB2, HA or in other genes) in our cases of ANE. The identified mutations do not belong to the category of mutations known to alter the virulence of the virus, cause strong drug resistance or reverse the effects of the premature STOP codon in the PB1-F2 gene of pandemic H1N1. Therefore, we can assume that the causative virus in our severe cases is not different from the virus circulating in the current season and is the virus used for the available vaccines. 

Acute necrotizing encephalopathy is a rare and very severe neurological disorder characterised by inflammation and necrosis in the brain. The global incidence of ANE is unknown, and most cases have been described in Asia, with a peak between age 18 months and 6 years [3]. The pathophysiology would result from a ‘cytokine storm’ in response to the viral infection, with increased vascular permeability, neuronal cytotoxicity and eventually neuronal cell death [3]. There are no data to suggest that the influenza A(H1N1) virus has a direct neurotoxicity effect based on findings from pathological and cerebrospinal fluid investigations [3] that we observed in our patients. There is no consensus on the best treatment strategy for ANE [9]. Anecdotally, different drugs, including high-dose steroids [10], plasma exchange [11], anakinra [12] and tocilizumab [13] have been used. Of our three most severe cases, the best outcome was observed in the child who received aggressive immunomodulation within 24 h of the onset of illness. Further studies are needed to define optimal treatment strategies based on higher level of evidence.

Interim 2023/24 A(H1N1)pdm09 influenza vaccine effectiveness in children was high (62–85%) in Canada [14] and Europe [15]. Although the risk for unfavourable outcomes in children with influenza is known, influenza vaccination coverage in children in Italy remains low (less than 10% in children under 4 years nationwide; ca 12% in the Tuscany region). The Italian Ministry of Health, following recommendations from the World Health Organization, recommends influenza vaccination in all children aged 6 months to 6 years. A poor influenza vaccine uptake could be the consequence of parental beliefs that influenza is a not-serious illness [16] and paediatricians’ belief that influenza vaccine has low effectiveness against mild to moderate illness [17]. However, influenza vaccine reduces the risk of critical illness in children [2], even in seasons when vaccine-mismatched influenza viruses circulate. 

## Conclusion

In summary, we observed an outbreak of life-threatening influenza A(H1N1)pdm09 illness in young children, resulting in a range of outcomes including severe neurological sequelae and death. Overall, these observations amounted to 3% of all patients hospitalised for fever and respiratory or neurological symptoms at our institution. We urge public health authorities and decisionmakers in the paediatric scientific community to join efforts to implement widespread influenza vaccination in young children.
